# Identification of genomic instability related lncRNA signature with prognostic value and its role in cancer immunotherapy in pancreatic cancer

**DOI:** 10.3389/fgene.2022.990661

**Published:** 2022-09-02

**Authors:** Xiaole Zhu, Rong Yu, Yunpeng Peng, Yi Miao, Kuirong Jiang, Qiang Li

**Affiliations:** ^1^ Pancreas Center, First Affiliated Hospital of Nanjing Medical University, Nanjing, Jiangsu, China; ^2^ Pancreas Institute, Nanjing Medical University, Nanjing, Jiangsu, China

**Keywords:** genome instability, somatic mutation, pancreatic cancer, long non-coding RNA, prognostic signature, tumor immune environment

## Abstract

**Background:** Increasing evidence suggested the critical roles of lncRNAs in the maintenance of genomic stability. However, the identification of genomic instability-related lncRNA signature (GILncSig) and its role in pancreatic cancer (PC) remains largely unexplored.

**Methods:** In the present study, a systematic analysis of lncRNA expression profiles and somatic mutation profiles was performed in PC patients from The Cancer Genome Atlas (TCGA). We then develop a risk score model to describe the characteristics of the model and verify its prediction accuracy. ESTIMATE algorithm, single-sample gene set enrichment analysis (ssGSEA), and CIBERSORT analysis were employed to reveal the correlation between tumor immune microenvironment, immune infiltration, immune checkpoint blockade (ICB) therapy, and GILncSig in PC.

**Results:** We identified 206 GILnc, of which five were screened to develop a prognostic GInLncSig model. Multivariate Cox regression analysis and stratified analysis revealed that the prognostic value of the GILncSig was independent of other clinical variables. Receiver operating characteristic (ROC) analysis suggested that GILncSig is better than the existing lncRNA-related signatures in predicting survival. Additionally, the prognostic performance of the GILncSig was also found to be favorable in patients carrying wild-type KRAS, TP53, and SMAD4. Besides, a nomogram exhibited appreciable reliability for clinical application in predicting the prognosis of patients. Finally, the relationship between the GInLncSig model and the immune landscape in PC reflected its application value in clinical immunotherapy.

**Conclusion:** In summary, the GILncSig identified by us may serve as novel prognostic biomarkers, and could have a crucial role in immunotherapy decisions for PC patients.

## Introduction

Pancreatic cancer is one of the deadliest cancers, ranking as the fourteenth most common cancer and the seventh leading cause of cancer mortality worldwide. Due to the lack of obvious early symptoms, PC usually presents at an advanced stage, which results in a 5-years survival rate as low as 6% (ranging from 2% to 9%) (McGuigan et al., 2018). Despite the great advances in surgery, chemotherapy, and radiotherapy for PC that have been made in the past few years, long-term survival and prognosis remain terrible, with more than 80 percent of patients facing recurrence after resection (Garrido-Laguna and Hidalgo, 2015). More recently, a large number of previous studies have analyzed the relationship between the expression of molecular markers and clinicopathology and long-term survival in the molecular mechanism of PC. However, their impact on patient early diagnosis and treatment is still limited (Garcea et al., 2005). Therefore, searching for new prognostic markers that can predict the poor outcome of patients may become the target of intervention, and provide new treatment strategies for the treatment of PC.

Genomic instability refers to an increased tendency of the genome to acquire mutations, which is typically conferred by some mechanism dysfunction, such as DNA damage repair, DNA replication, transcription, and so on. Genomic instability is a hallmark of cancer and is related to cancer initiation and progression (Duijf et al., 2019). In addition, genome stability status is also associated with survival and can be used as a prognostic marker for cancer patients (Gupta et al., 2018). Long non-coding RNAs (lncRNAs) are arbitrarily considered as non-protein coding transcripts over 200 nucleotides in length (Ma et al., 2014). There is increasing evidence suggesting that lncRNAs are involved in a variety of biological processes and play a critical role in genome regulation (Mercer et al., 2009; Rinn and Chang, 2012; Ma et al., 2014). Noticeably, the dysregulation of lncRNAs has been established to be associated with many complex diseases, including cancers (Gibb et al., 2011; Spizzo et al., 2012; Cheetham et al., 2013). Many lncRNAs are abnormally expressed in tumor tissues, which have been considered oncogenes, such as MALAT1 (Wang et al., 2017), HOTAIR (Troiano et al., 2017), H19 (Zhang et al., 1993), and MEG3 (Braconi et al., 2011). The main function of lncRNA is to regulate gene expression and indicate the tumor status better than the protein-coding RNAs, so it can be used as a novel biomarker with diagnostic and prognostic significance (Hauptman and Glavac, 2013). Currently, several lncRNA signatures have been developed in various cancers to predict patient prognosis with great predictive performance, including lung cancer (Lin et al., 2018), head and neck squamous cell carcinoma (Diao et al., 2019), ovarian cancer (Zhou et al., 2016) and breast cancer (Fei et al., 2018; Tang et al., 2019). Recently, Lee et al. (2016) analyzed a non-coding RNA activated by DNA damage (or NORAD) and maintained genomic stability by isolating PUMILIO protein. Hu et al. reported that GUARDIN, as a p53-responsive lncRNA, kept genomic integrity under both stable and exposed status (Hu et al., 2018). These results demonstrated the important role of lncRNAs in maintaining genomic stability, but the lncRNAs associated with genomic instability need to be further explored.

In addition, studies have shown that immune cells act as tumor inhibitors or tumor promoters and may function as important players in the tumor immune microenvironment (TIME). Genomic instability has been termed as a promising indicator for predicting responsiveness to immune checkpoint blockade based on numerous researches.

Therefore, we constructed a GILncSig to investigate whether the lncRNA signature could reflect the tumor immune microenvironment, and serve as an effective prognostic predictor for patients with PC.

## Methods

### Availability of data and materials

The clinical information, RNA-seq expression data, lncRNA transcriptional profiles, and somatic mutation information of patients with PC were obtained from TCGA project (https://cancergenome.nih.gov/). A total of 171 TCGA PC patients with lncRNA expression profiles somatic mutations, survival information, and clinical features were utilized in our study. TCGA patients with PC were divided into an 84-sample training set and an 87-sample testing set. The training set was used to identify the prognostic lncRNA signature and establish the prognostic risk model, while the testing set was used to independently validate its prognostic value.

### Identification of genomic instability-associated lncRNAs

**FIGURE 1:**
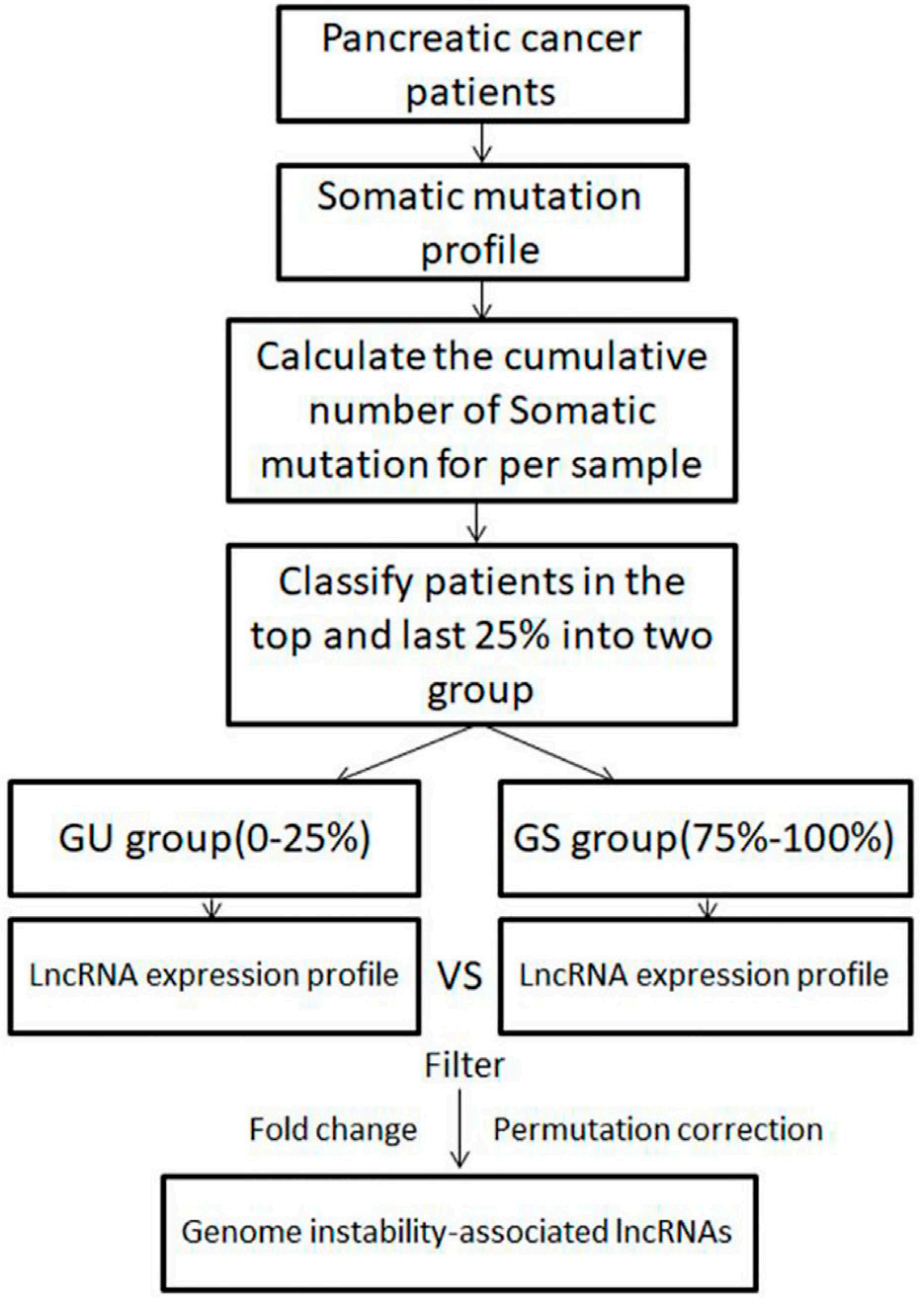
Computational framework of genomic instability-associated lncRNAs detection.

To identify genomic instability-associated lncRNAs, a computational framework was constructed based on the lncRNAs expression profiles and somatic mutation profiles of PC patients. As shown in Figure 1, the cumulative number of somatic mutations per sample was calculated and arranged in descending order. The first 25% of patients were defined as the genomic instability group (GU group), and the last 25% were defined as the genomic stability group (GS group). Then compared the expression profiles of lncRNAs between the GU group and GS group by the significance analysis of microarrays (SAM) method. The differentially expressed lncRNAs screened out by the filter of fold change and permutation correction were defined as GILnc (fold change >1.5 or <0.67 and false discovery rate (FDR) adjusted *p* < 0.05).

### Functional enrichment analysis

We calculated the Pearson correlation coefficient to evaluate their correlation by using paired lncRNA and mRNA expression profiles and then established a lncRNA-mRNA co-expression network. Gene Ontology (GO) and Kyoto Encyclopedia of Genes and Genomes (KEGG) enrichment analyses of the co-expressed protein-coding genes with prognostic lncRNAs were performed to predict the biological function of the differentially expressed lncRNAs using clusterProfiler software in R-version 3.5.2 (Yu et al., 2012).

### Tumor immune-related analysis

To reflect the characteristics of the tumor immune microenvironment, the R package “ESTIMATE” was utilized to calculate Scores of immune and stromal cells. Immune infiltration information containing each tumor sample’s immune cell fraction was obtained from Tumor Immune Estimation Resource (TIMER) (https://cistrome.shinyapps.io/timer/). The correlation of tumor immune cell infiltrating with prognostic risk signature was further analyzed. We selected six key genes of immune checkpoint blockade–related genes in PC to investigate the potential role of a lncRNA-based signature in ICB therapy of PC.

### Statistical analysis

We carried out a univariate regression analysis to determine the relationship between the expression level of lncRNAs and the overall survival of the training set. Those lncRNAs with a *p*-value less than 0.05 were considered as the candidate prognostic lncRNAs of PC whose expression levels were significantly associated with the overall survival of PC patients. To assess the contribution of that candidate lncRNA as an independent prognostic factor for survival, a multivariate Cox regression analysis was further performed. A *p* value less than 0.05 was considered significant. A prognostic risk score model of GILncSig was constructed based on the expression level of lncRNAs and multivariate Cox regression coefficient to predict the prognosis of patients with PC as follows: GILncSig (patients) = $\overset{\text{n}}{\underset{\text{i}=1}{\sum }}\text{coefficient}\left(\text{lncRNAi}\right)$ * expression(lncRNAi) . In our formula, GILncSig (patients) is the prognostic risk score for PC patients. lncRNAi is each prognostic lncRNAs. Coefficient (lncRNAi) represents the corresponding coefficient of multivariate Cox regression analysis, and expression (lncRNAi) is the expression level of lncRNAi.

According to the above formula, the lncRNA expression-based risk scores for PC patients could be calculated and divided patients into high-risk and low-risk groups with the cutoff of the median risk score from the training set. Kaplan-Meier survival curves were utilized to estimate the survival rate of the different patient groups, and the survival differences between the high-risk group and low-risk group were assessed by the log-rank test. Time-dependent ROC analysis for overall survival was used to assess the performance of the prognostic risk model for time-dependent disease outcomes. Multivariate Cox regression and stratified analysis were performed to determine whether the GILncSig was independent of other clinical variables. Hazard ratio (HR) and 95% confidence intervals (CI) were estimated by Cox proportional hazards regression model. A nomogram was built in the training set to predict the 1-, 2-, and 3-years survival based on the results of multivariate cox regression analysis by R “rms” and “survival” package and applied to the testing set and the entire TCGA set for verification. The corrected plot was used to assess the prognostic accuracy of the nomogram. All statistical analyses were performed using R software and Bioconductor.

## Result

### Identification of genome instability-associated lncRNAs in patients with pancreatic cancer

**FIGURE 2:**
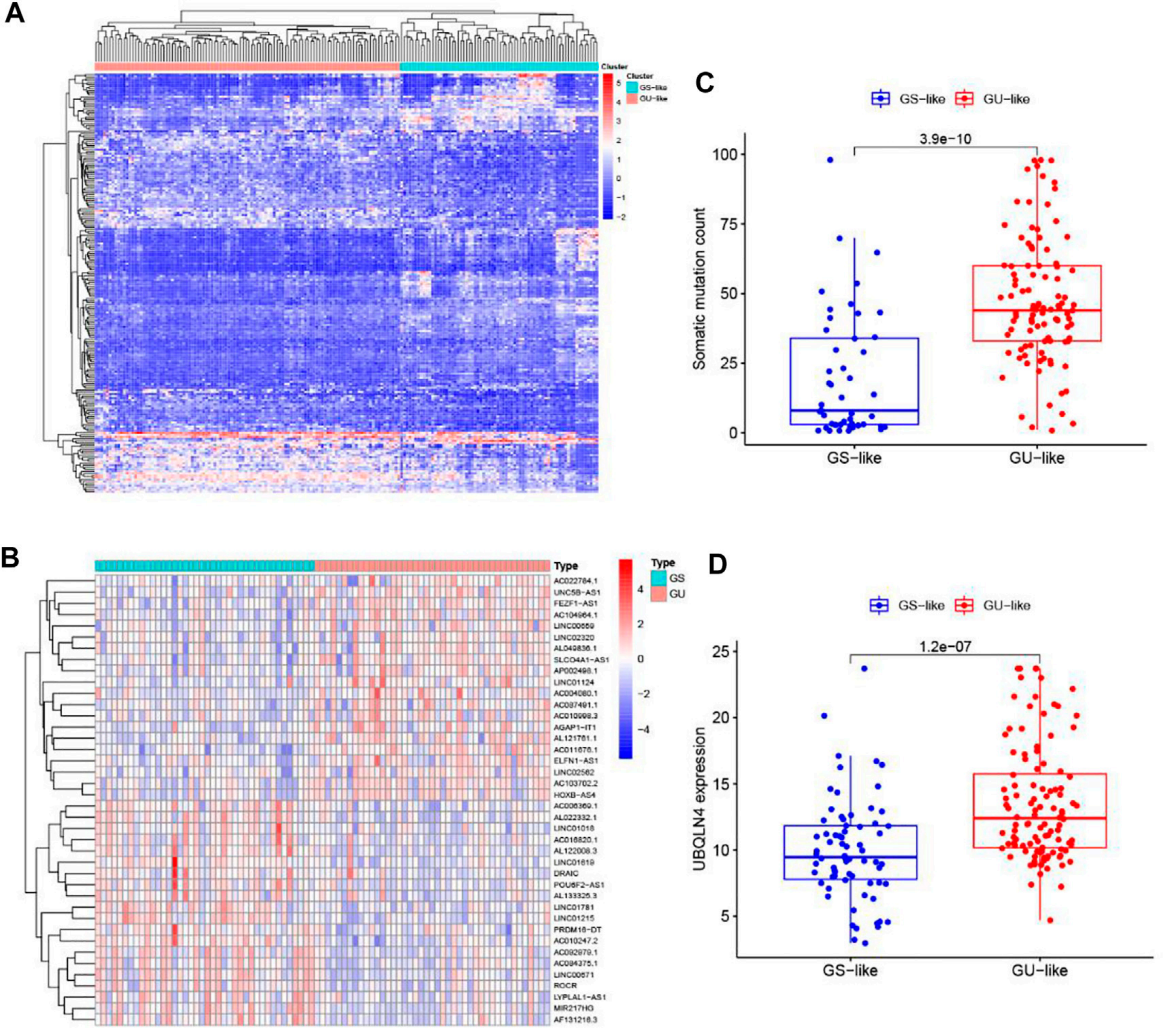
Identification of genome instability-related lncRNAs in patients with pancreatic cancer. **(A)** Unsupervised clustering analysis of the lncRNA expression profiles in the GU group and GS group. **(B)** Unsupervised clustering analysis of 171 patients with pancreatic cancer according to the differential expression patterns of 206 GILnc. **(C)** Boxplots of somatic mutations count in the GU-like group and GS-like group. **(D)** Boxplot of the expression level of UBQLN4 in the GU-like group and GS-like group.

To detect the potential Genome instability-related lncRNAs, the cumulative number of somatic mutations in each patient with PC was calculated from TCGA. The first 25% (*n* = 43) and the last 25% (*n* = 40) patients were classified into the GU group and GS group by the descending order of cumulative number. Then the lncRNA expression profiles in the GU group and GS group were analyzed by unsupervised clustering, the result shows that a total of 206 lncRNAs were found to be significantly differentially expressed (Figure 2A). All patients with PC in TCGA were divided into GU-like group and GS-like group by unsupervised hierarchical clustering analysis based on the expression levels of the 206 differentially expressed lncRNAs. The cumulative number of somatic mutations was higher in the GU-like group and lower in the GS-like group (Figure 2B). As shown in Figure 2C, more mutated genes exist in the GU-like group (*p* < 0.001, Mann-Whitney *U* test). As the UBQLN4 gene is one of the driving factors of gene instability, the expression level of the UBQLN4 gene in the GU-like group and GS-like group was compared. The results showed that there was a significant difference in the expression level of UBQLN4 between the two groups, and the expression level of UBQLN4 in the GU-like group was significantly higher than that in the GS-like group. (*p* < 0.001, Mann–Whitney *U* test, Figure 2D).

**FIGURE 3:**
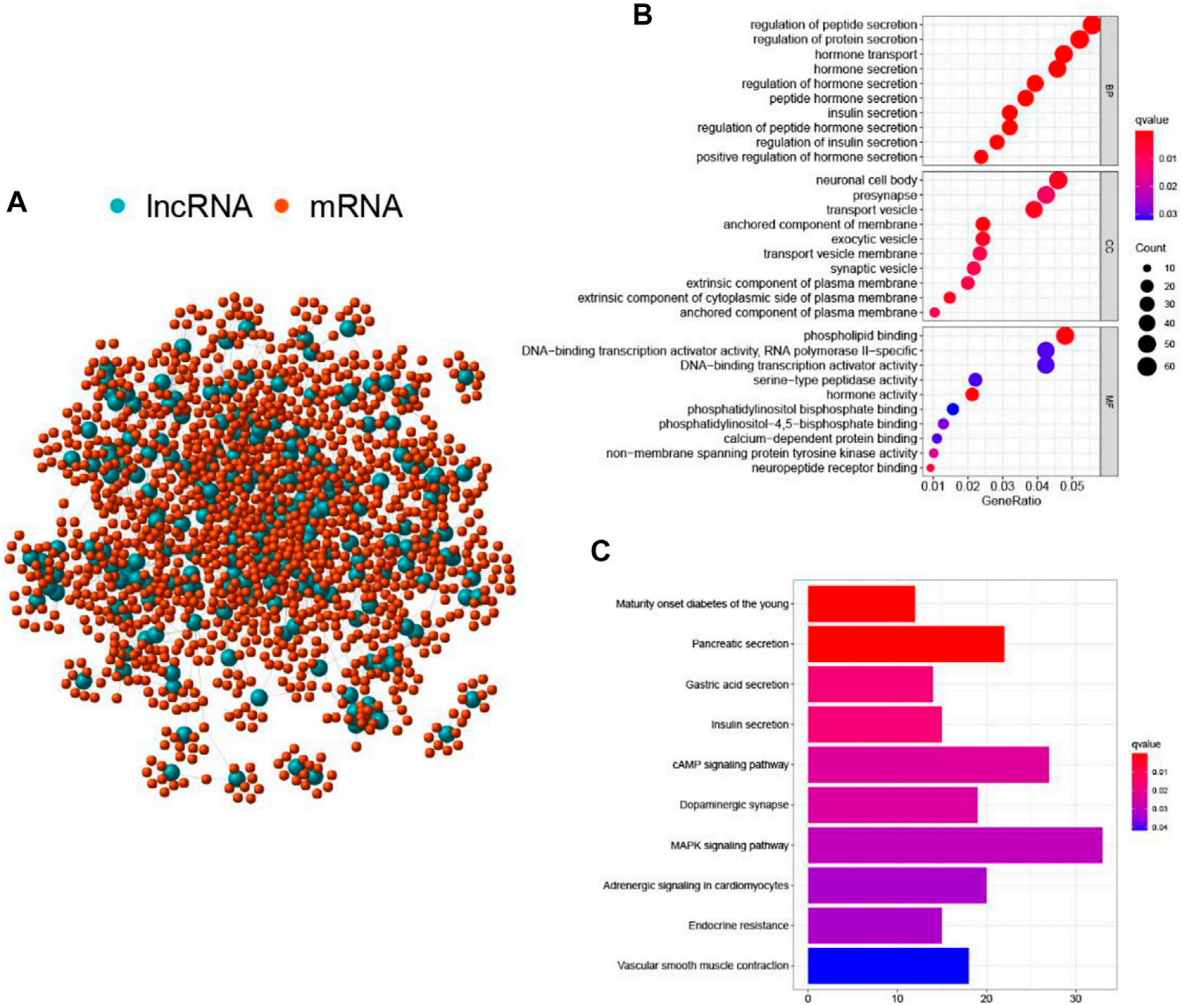
Functional annotations of GILnc in patients with pancreatic cancer. **(A)** Co-expression network of GILnc and mRNAs. The blue and red circles represented the LncRNAs and protein-coding mRNAs, respectively. Functional enrichment analysis of GO biological process **(B)** and KEGG pathway **(C)** for the co-expressed protein genes with lncRNAs.

To better understand the biological significance of the 206 differentially expressed lncRNAs, functional enrichment analysis was performed to predict potential functions. We selected the protein-coding genes (PCGs) most related to the expression of each lncRNA to construct a lncRNA-mRNA co-expression network (Figure 3A). According to the enriched results of the lncRNA-correlated PCGs, GO biological process (e.g., cellular component (CC), DNA binding in the molecular function (MF), and metabolism in the biological process (BP)) and KEGG pathway (e.g., MAPK signaling pathway, cAMP signaling pathway, Pancreatic secretion, and Endocrine resistance) were annotated to be associated with genome instability (Figures 3B,C). Based on the above results, it is considered that the 206 lncRNAs were involved in the genomic instability-related biological process, and their altered expression may destruct the genomic stability of cells. Therefore, the 206 differentially expressed lncRNAs were recognized as candidate lncRNAs with genomic instability in PC.

## Acquisition of a genomic instability-associated lncRNA prognostic signature from the training set

**FIGURE 4:**
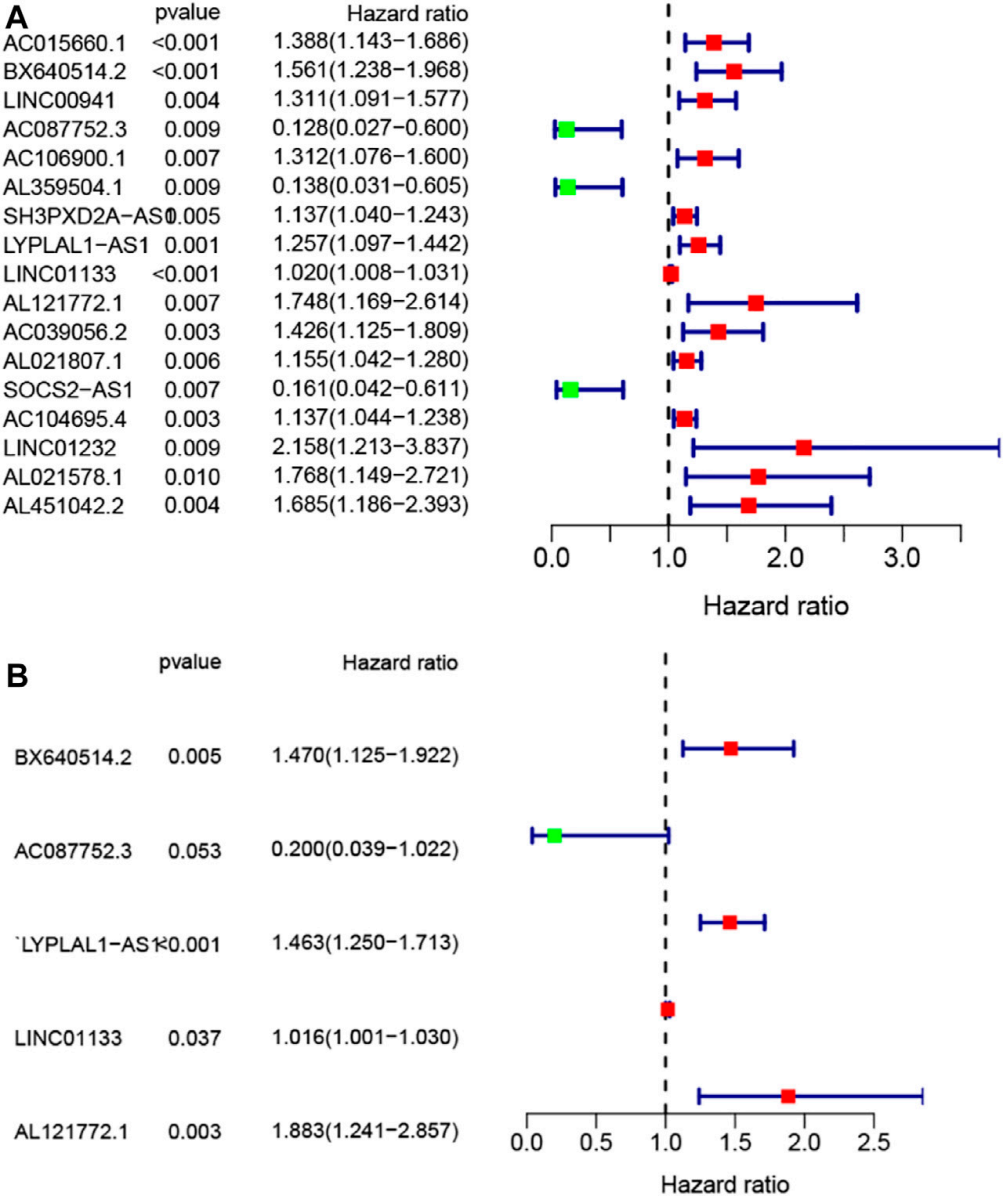
Construction of the genomic instability-associated lncRNA prognostic signature from the training set. **(A)** Forest plot of 17 candidate prognostic LncRNAs associated with pancreatic cancer patients’ overall survival based on univariate Cox regression analyses. **(B)** Forest plot of five candidate prognostic LncRNAs associated with pancreatic cancer patients’ overall survival based on stepwise multivariate Cox proportional hazard regression.

**FIGURE 5:**
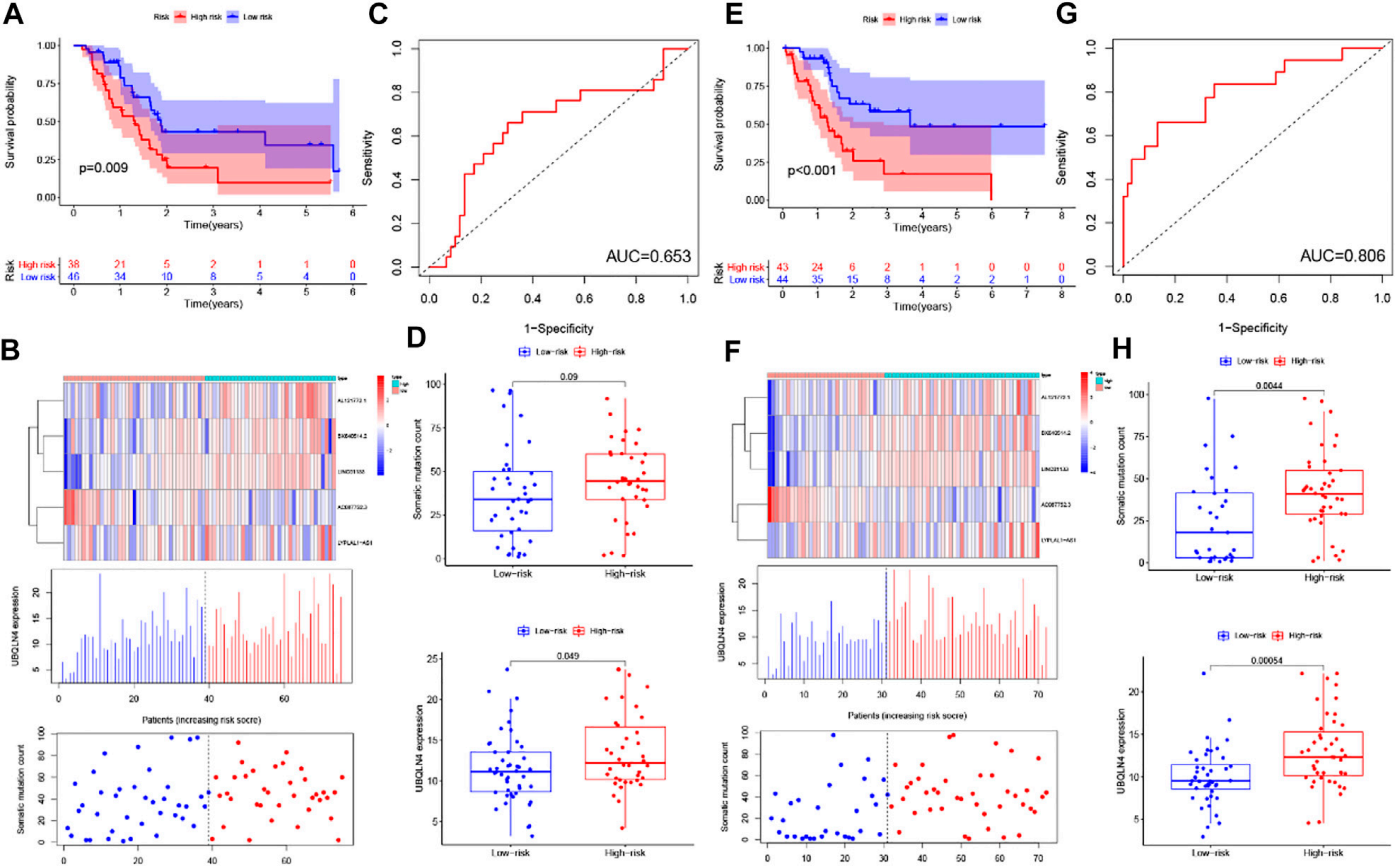
Identification and validation of the GILncSig for outcome prediction in patients with pancreatic cancer in the training set and testing set. **(A,E)** Kaplan–Meier survival curves of patients in the high- and low-risk groups are separated by the median GILncSig score in the training set **(A)** and testing set **(E)**. **(B,F)** LncRNA expression patterns and the distribution of somatic mutation and UBQLN4 expression with increasing GILncSig score in the training set **(B)** and testing set **(F)**. **(C,G)** Time-dependent ROC curves for 1-year survival prediction of the GILncSig in the training set **(C)** and testing set **(G)**. **(D,H)** Boxplots of comparison of the somatic mutation counts and the UBQLN4 expression between the high- and low-risk groups in the training set **(D)** and testing set **(H)**.

To screen out the prognostic lncRNAs with independent value, we performed a univariate Cox proportional hazard regression analysis to analyze the relationship between expression levels of 206 GIlncRNA and OS in the training set, 17 candidate prognostic lncRNAs were found to be significantly associated with the prognosis of PC patients (Figure 4A). Furthermore, multivariate Cox proportional hazards regression was used to analysis on 17 candidate prognostic lncRNAs. Based on the multiCox model (Figure 4B), 4 of 17 candidate lncRNAs including AL121772.1, BX640514.2, LINC01133, and LYPLAL1-AS1 were found to retain their prognostic significance and thus were identified as independent prognostic lncRNAs (*p* < 0.05). All of the four lncRNAs (AL121772.1, BX640514.2, LINC01133, and LYPLAL1-AS1) with positive coefficients tended to be prognostic risk factors and their high expression were associated with shorter survival. One lncRNAs (AC087752.3) having negative coefficients was shown to be a protective factor whose high expression level was closed associated with longer survival. A risk score model of GILncSig based on the results of the multivariate Cox analysis regression coefficients was generated to predict the outcome of PC patients as follows: GILncSig = (−1.61 × expression value of AC087752.3) + (0.63 × expression value of AL121772.1) + (0.39× expression value of BX640514.2) + (0.02 × expression value of LINC01133) + (0.38× expression value of LYPLAL1-AS1). According to the GILncSig model, the prognostic risk score was computed for each patient in the training set. Using the median risk score as the cutoff point, all patients in the training set were classified into a high-risk group (*n* = 38) and a low-risk group (*n* = 46). The Kaplan-Meier analysis indicated that the overall survival was significantly different between the two risk groups and patients in the low-risk subgroup had markedly longer overall survival than those in the high-risk group (*p* = 0.009, log-rank test, Figure 5A). The time-dependent receiver operating characteristic (ROC) curves analysis for GIlncRNA prognostic model achieved an area under the curve (AUC) of 0.653 at 1 year of overall survival (Figure 5C). These results demonstrated the GIlncRNA had better prognosis prediction performance in patients with PC. Then we ranked the risk scores of patients in the training set. Figure 5B showed the expression pattern of the five Independent prognostic lncRNAs, the expression level of UBQLN4, and the count of somatic mutations. We found that for patients with high-risk scores, the expression levels of four risk lncRNAs(AL121772.1, BX640514.2, LINC01133, LYPLAL1-AS1) were up-regulated, while one protective lncRNA (AC087752.3) was expressed at a low level. In contrast, these prognostic lncRNAs expressed the opposite patterns in patients with low-risk scores. Similarly, there were significant differences in UBQLN4 expression levels between the high-risk group and low-risk group (*p* = 0.049, Mann–Whitney *U* test; Figure 5D). Moreover, Figure 5D also revealed that the number of somatic mutations in the high-risk group was slightly higher than those in the low-risk group (*p* = 0.09, Mann–Whitney *U* test; Figure 5D).

### Validation of GILncSig in the testing set and entire the cancer genome atlas set

To confirm our findings, the prognostic performance of the GILncSig was further evaluated in the testing set. Patients in the testing set were divided into the high-risk group (*n* = 43) and the low-risk group (*n* = 44) by using the same GILncSig and cutoff value deriving from the training set. Kaplan-Meier curves showed that there was a significant difference in overall survival between the high-risk group and the low-risk group, and the overall survival of the high-risk group was much lower than the low-risk group (*p* < 0.001, log-rank test, Figure 5E), which were similar to those observed in the training set. Validation of the GILncSig in the testing set of 87 patients produced a ROC with an AUC of 0.806 at 1 year (Figure 5G). Figure 5F shows how the expression level of GILncSig, the count of somatic mutation, and the expression level of UBQLN4 in the testing set change with the increasing score. The analysis indicated that Somatic mutation counts and the expression level of UBQLN4 were significantly higher in the high-risk group as compared with those in the low-risk group (*p* = 0.0044, *p* = 0.00054, Mann-Whitney *U* test; Figure 5H).

**FIGURE 6:**
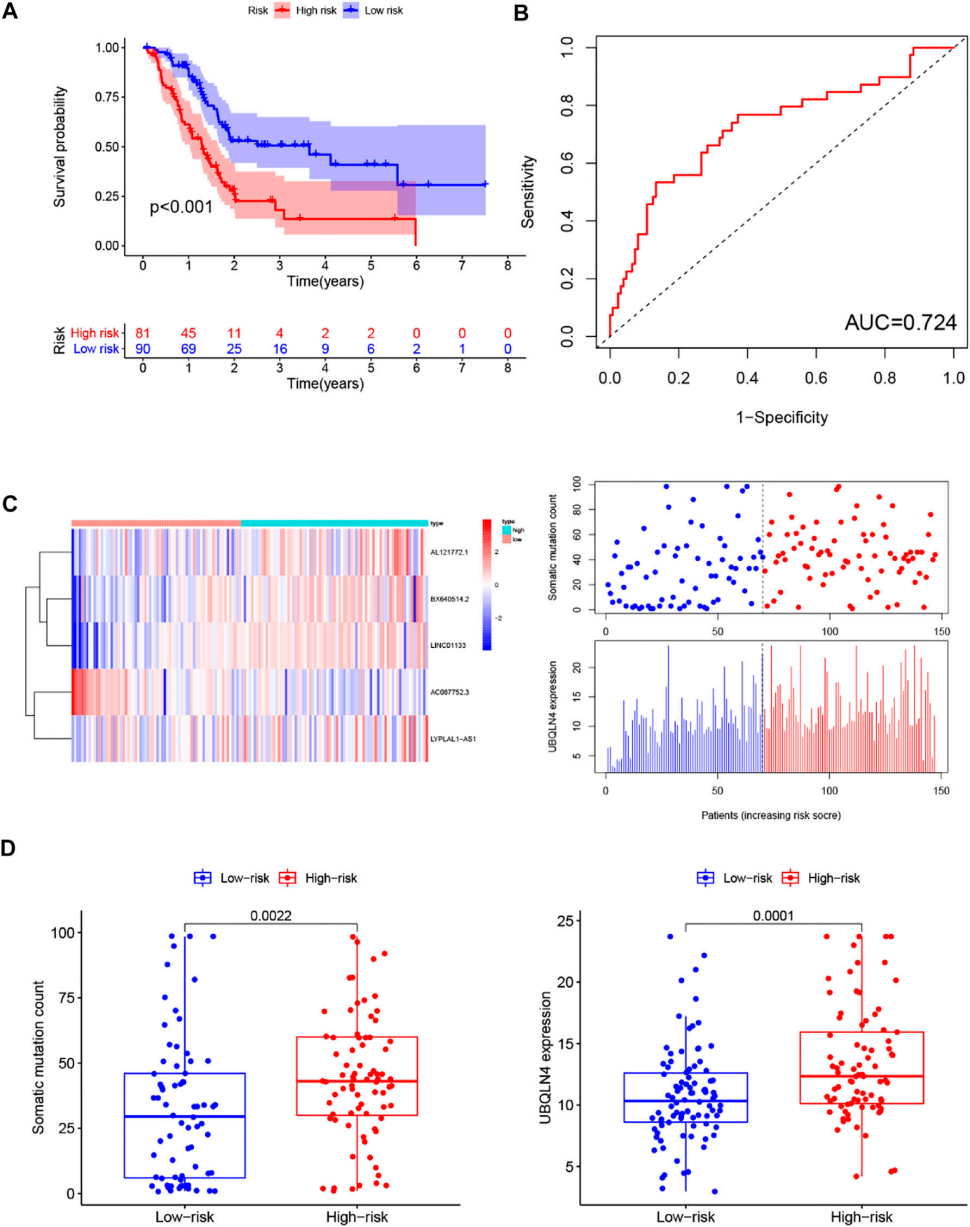
Predictive performance evaluation of the GILncSig in patients with pancreatic cancer in the TCGA set. **(A)** Kaplan–Meier survival curves of patients in the high- and low-risk groups separated by the median GILncSig score in the TCGA set. **(B)** LncRNA expression patterns and the distribution of somatic mutation and UBQLN4 expression with increasing GILncSig score in the TCGA set. **(C)** Time-dependent ROC curves for 1-year survival prediction of the GILncSig in the TCGA set. **(D)** Boxplots of comparison of the somatic mutation counts and the UBQLN4 expression between the high- and low-risk groups in the TCGA set.

Similar results were observed when the prognostic performance of the GILncSig was further used to the entire TCGA set. Like the training and testing set, the GIlncRNA was able to stratify 171 PC patients of the entire TCGA set into the high-risk group (*n* = 81) and low-risk group (*n* = 90) with obviously different overall survival (*p* < 0.001, log-rank test, Figure 6A). The AUC of time-dependent ROC analysis for overall survival in the entire TCGA set was 0.724 (Figure 6B). The expression of GILncSig, somatic mutation counts, and UBQLN4 expression level of PC patients in the TCGA set was presented in Figure 6C, which were similar to those observed in the training set and testing set. The counts of somatic mutations in the high-risk group were significantly higher than that in the low-risk group (*p* = 0.0022, Mann-Whitney *U* test, Figure 6D), as was the expression level of UBQLN4 (*p* = 0.0001, Mann-Whitney *U* test, Figure 6D).

### Comparison of the GILncSig and other lncRNA-related predictive signatures for survival prediction

**FIGURE 7:**
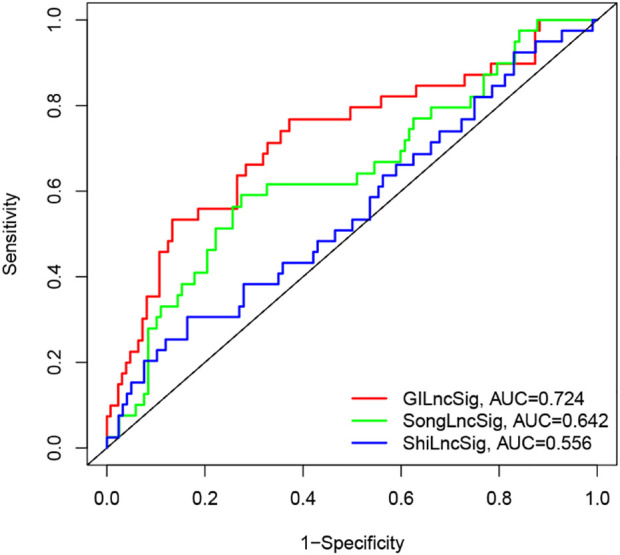
The ROC analysis for 1-year survival prediction of the GInLncSig and the other two existing signatures (SongLncSig, ShiLncSig), respectively.

Recently, two lncRNA-related signatures were reported to predict the prognosis of PC patients. Therefore, we further compared the prognostic value of our GILncSig to that of different lncRNA-associated signatures for predicting outcomes: the five-lncRNA signature derived from Song’s study (hereinafter referred to as SongSig) (Song et al., 2018) and the three-lncRNA signature derived from Shi’s study (hereinafter referred to as ShiSig) (Shi et al., 2018). Utilizing the same TCGA patient set. Then we performed the time-dependent ROC analysis and calculated the area under the ROC curves to compare the prediction performance between the GILncSig and other two existing lncRNA-related signatures in the entire TCGA set. The result demonstrated that the AUC at 1 year of overall survival for the GILncSig is 0.724, which was significantly higher than that of SongSig (AUC = 0.642) and ShiSig (AUC = 0.556) (Figure 7). For this reason, we believed that the GILncSig had better prognostic power than those two lncRNA-related signatures.

## Independence of prognostic value of the GIlncRNA from other clinical variables

**FIGURE 8:**
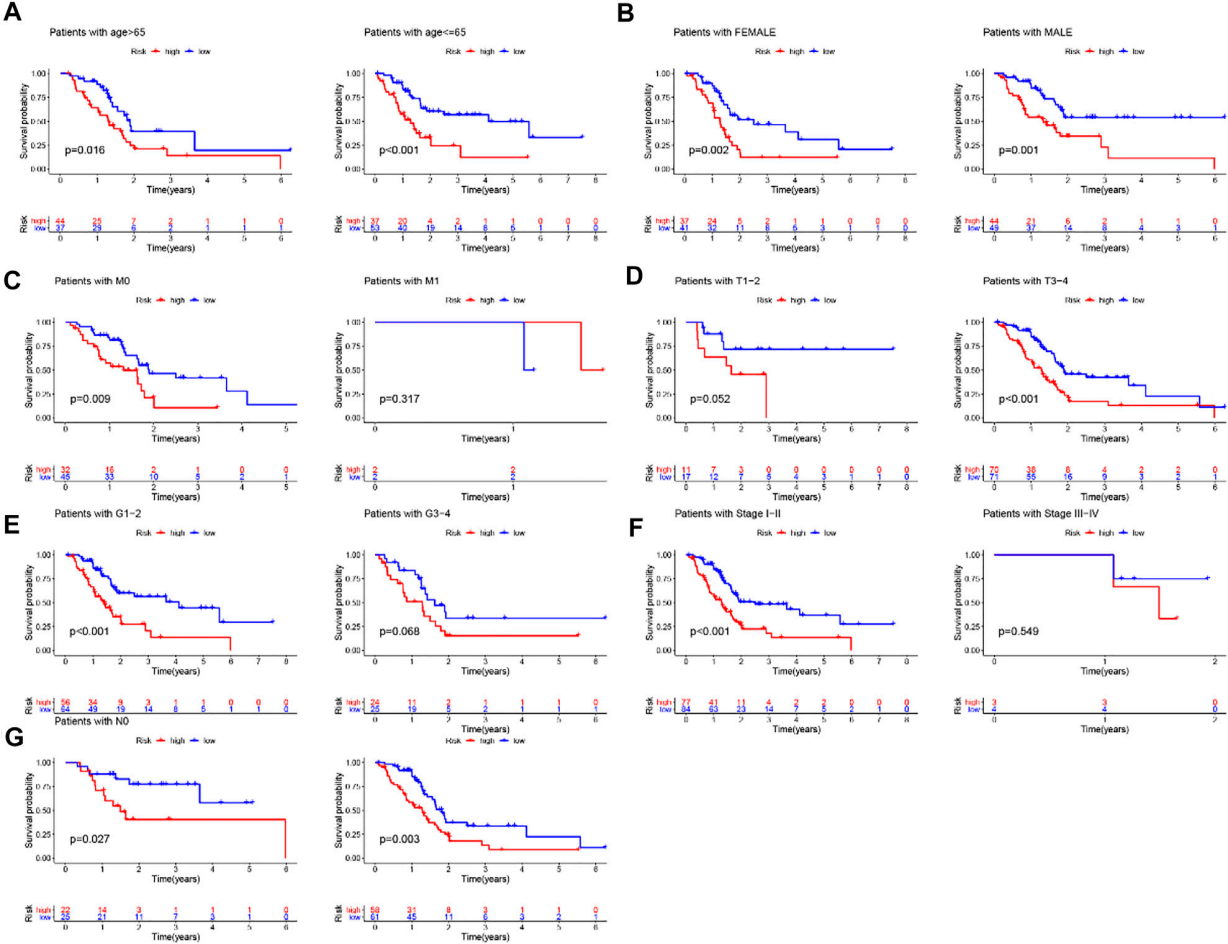
Stratification analysis by age, gender and pathological grade and stage. **(A)** Kaplan-Meier curve analysis of overall survival in low-/high-risk groups for age. **(B)**Kaplan-Meier curve analysis of overall survival in low-/high-risk groups for gender. **(C,D,G)** Kaplan-Meier curve analysis of overall survival in low-/high-risk groups based on tumor size, lymph node metastasis and distant metastasis. **(E)** Kaplan-Meier curve analysis of overall survival in low-/high-risk groups for the grade. **(F)** Kaplan-Meier curve analysis of overall survival in low-/high-risk groups for stage.

To determine whether the prognostic value of the GIlncRNA was independent of other clinical variables. Multivariate Cox regression analysis was performed in each patient set using prognostic risk score, age, gender, pathological grade, and stage. Results from multivariate Cox analysis revealed that the GIlncRNA was significantly associated with overall survival in each set when adjusted for age, gender, pathological grade, and stage (Table 1). At the same time, we also observed that age, gender, pathological grade, and stage were different in the multivariate analysis significantly. So we further performed data stratification analysis according to age and gender, pathological grade, and stage. According to age, PC patients could be stratified into an old patient group (age >65, *n* = 81) and a young patient group (age <=65, *n* = 90). The GIlncRNA could subdivide each age group into a high-risk group and a low-risk group. There was significantly different overall survival between the high-risk group and low-risk group in each age group. (log-rank test *p* = 0.016 for the old patient group and log-rank test *p* < 0.001 for the young patient group) (Figure 8A). Next, all patients were also stratified by gender. The overall survival of patients in the low-risk group was significantly longer than that of patients in the high-risk group by analysis of the results. (log-rank test *p* = 0.002 for the female group; log-rank test *p* = 0.001 for the male group; Figure 8B). In addition, all patients in the entire TCGA set were grouped according to tumor size, lymph node metastasis, and distant metastasis. Each group was further separated into a high-risk group and a low-risk group by the GIlncRNA, and the difference in overall survival between the two groups was compared. As shown in Figure 8, except for the metastatic group (M1 group and T1-2 group), there were statistically significant differences in overall survival between the high-risk and low-risk groups in each group (*p* < 0.001 for T3-4 group, Figure 8D; *p* = 0.027 for N0 group, *p* = 0.003 for N1 group, Figure 8G; *p* = 0.009 for M0 group, *p* = 0.317 for M1 group, Figure 8C; log-rank test). Finally, the same analysis method was applied to the pathological grade and stage of patients. The results of the stratified analysis showed that the patients with high grades were divided into either a high-risk group (*n* = 24) with shorter survival or a low-risk group (*n* = 25) with longer survival (*p* = 0.068, log-rank test; Figure 8E). The patients in the low-grade group were similarly classified into two risk subgroups with significantly different survival times (*p* < 0.001, log-rank test; Figure 8E). Furthermore, patients with pathologic stage I or II were combined into an early-stage group (*n* = 161), and those with pathologic stage III or IV were combined into a late-stage group (*i* = 7). The GIlncRNA divided the early-stage group and the late-stage group into a high-risk group and a low-risk group respectively. The overall survival was significantly different between the two groups in the early-stage group (*p* < 0.001, log-rank test; Figure 8F). Nevertheless, the difference in overall survival between the two groups was not significant probably due to the limited sample size in the late group (*p* = 0.549, log-rank test; Figure 8F). Taken together, these results indicated that the GILncSig was an independent prognostic factor associated with overall survival in PC patients.

**TABLE 1:** Univariate and Multivariate Cox regression analysis of the GILncSig and clinical features for the independent prognostic significance in different patient datasets.

Variables	Univariable model				Multivariable model			
	HR	HR.95L	HR.95H	p value	HR	HR.95L	HR.95H	p value
TCGA set								
age	1.027207	1.005871	1.048994	0.012189	1.023761	1.001864	1.046137	0.033272
gender	0.873723	0.577194	1.322592	0.523359				
grade	1.391989	1.040839	1.861608	0.025759	1.250423	0.931461	1.678608	0.136906
stage	1.365182	0.936063	1.991023	0.105923				
riskScore	1.030134	1.014894	1.045604	9.46E-05	1.02821	1.013716	1.042912	0.000123
Testing set								
id	HR	HR.95L	HR.95H	pvalue	HR	HR.95L	HR.95H	pvalue
age	1.02934	1.000879	1.058611	0.04324	1.02934	1.000879	1.058611	0.04324
gender	1.051846	0.60187	1.838237	0.859146				
grade	1.341221	0.926544	1.941486	0.119783				
stage	1.341823	0.799856	2.251018	0.265316				
riskScore	1.029268	0.964336	1.098572	0.38557				
Training set								
id	HR	HR.95L	HR.95H	pvalue	HR	HR.95L	HR.95H	pvalue
age	1.026642	0.994416	1.059912	0.106125				
gender	0.768614	0.409857	1.4414	0.412039				
grade	1.454163	0.904621	2.337542	0.122089				
stage	1.439642	0.82667	2.507129	0.19794				
riskScore	1.026271	1.011035	1.041736	0.000679	1.02934	1.000879	1.058611	0.04324

### The prognostic significance of GILncSig is better than KRAS, TP53, and SMAD4 mutation status

**FIGURE 9:**
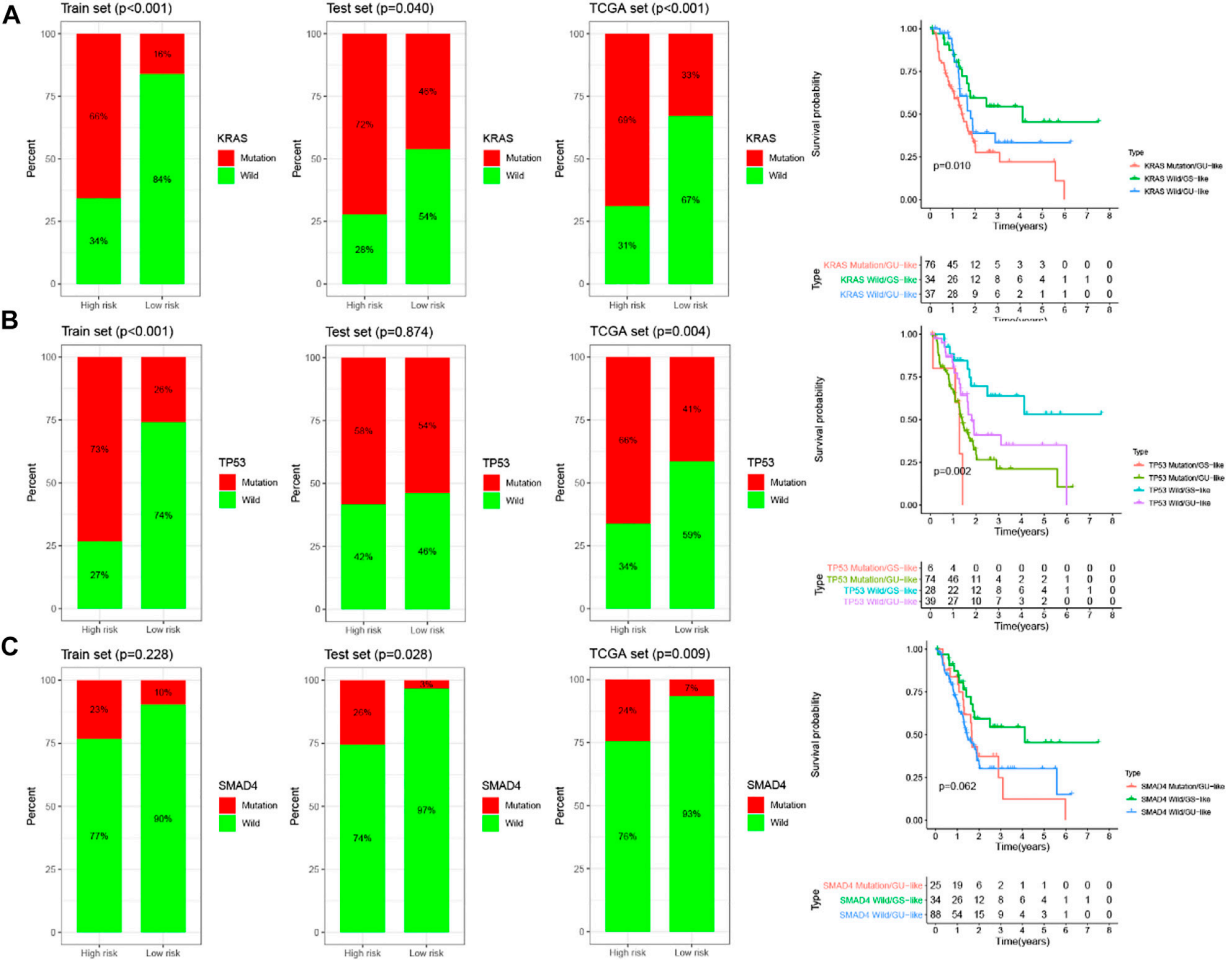
Relationship between the GILncSig and KRAS, TP53, SMAD4 mutation. The proportion of KRAS **(A)**, TP53 **(B)**, and SMAD4 **(C)** mutation in the high- and low-risk group in the training set, the testing set, and the TCGA set. Kaplan–Meier survival curves of overall survival for patients in groups divided based on KRAS **(A)**, TP53 **(B)**, SMAD4 **(C)** mutation status and the GILncSig.

KRAS, TP53 and SMAD4 were the most frequent mutant genes and associated with poor prognosis in PC. With this in mind, these three genes were included in the training set, testing set and TCGA set for analysis, respectively. Then further stratified analysis was performed based on the mutation status of KRAS, TP53 and SMAD4 by GILncSig. The analysis showed that the proportion of patients with KRAS, TP53, and SMAD4 mutations in the high-risk group was higher than that in the low-risk group to varying degrees in each set. For KRAS, 66% of the high-risk group had KRAS mutations, significantly higher than 16% of the low-risk group in the training set (chi-square test *p* < 0.001). In the testing set, 72% of the high-risk group had KRAS mutation, which was significantly higher than 46% of the low-risk group (chi-square test *p* = 0.040). In the entire TCGA set, 69% of KRAS mutation in the high-risk group was significantly higher than 33% in the low-risk group (chi-square test *p* < 0.001). These results suggest that GILncSig is closely related to the mutation state of the KRAS gene. Therefore, we applied GILncSig to patients with KRAS Wild type (KRAS Wild) and KRAS mutation type (KRAS mutation). Patients with KRAS Wild were divided into the low-risk group (KRAS Wild/GS-like) and high-risk group (KRAS Wild/GU-like), and patients with KRAS mutation were divided into the low-risk group (KRAS Wild/GS-like) and high-risk group (KRAS mutation/GU-like). Through comparative analysis, we found that the overall survival of the KRAS Wild/GS-like group was significantly different from that of the KRAS Wild/GU-like group and KRAS Wild/GU-like group, and patients in KRAS Wild/GS-like group had better prognosis (*p* = 0.01, log-rank test; Figure 9A). For TP53, as shown in Figure 9B, 73% of TP53 mutations in the high-risk group were significantly higher than 26% in the low-risk group in the training set (chi-square test *p* < 0.001). Similarly, in the TCGA set, the TP53 mutation in the high-risk group was higher than that in the low-risk group (high-risk group 66% versus low-risk group 41%, chi-square test *p* = 0.004). However, TP53 mutations were only slightly higher in the high-risk group than that in the low-risk group in the test set, and there was no significant difference between the two groups (high-risk group 58% versus low-risk group 54%, chi-square test *p* = 0.874). In consequence, we believe that TP53 status can be predicted according to the GILncSig risk score. Then patients with TP53 mutation and TP53 wild type were further divided into TP53 mutation high-risk group (TP53 mutation/GU-like), TP53 mutation low-risk group (TP53 mutation/GS-like), TP53 wild high-risk group (TP53 wild/GU-like), and TP53 wild low-risk group (TP53 wild/GS-like). Survival analysis showed that patients in the TP53 wild/GS-like group had longer survival than those in the TP53 wild/GU-like group, and the higher risk scores were associated with lower survival rates in TP53 wild subgroups (*p* = 0.002, log-rank test; Figure 9B). For SMAD4, it has similar results to KRAS and TP53. The patients in the training set, testing set and TCGA set were respectively divided into high-risk group and low-risk group by using GILncSig. In each set, the proportion of SMAD4 mutation in the high-risk group was significantly higher than that in the low-risk group (*p* = 0.228 for the training set; *p* = 0.028 for the testing set; *p* = 0.009 for TCGA set; chi-square test; Figure 9C). The patients with SMAD4 mutation type and SMAD4 wild type were further separated into SMAD4 mutation/GU-like group, SMAD4 mutation/GS-like, SMAD4 wild/GU-like group and SMAD4 wild/GS-like group. The results of the survival analysis showed that the overall survival among the groups was slightly different (*p* = 0.062, log-rank test; Figure 9C). Therefore, the above findings suggested that the GILncSig is superior to KRAS, TP53, and SMAD4 mutation status in prognosis.

## Development and validation of a nomogram for predicting survival in patients with pancreatic cancer

**FIGURE 10:**
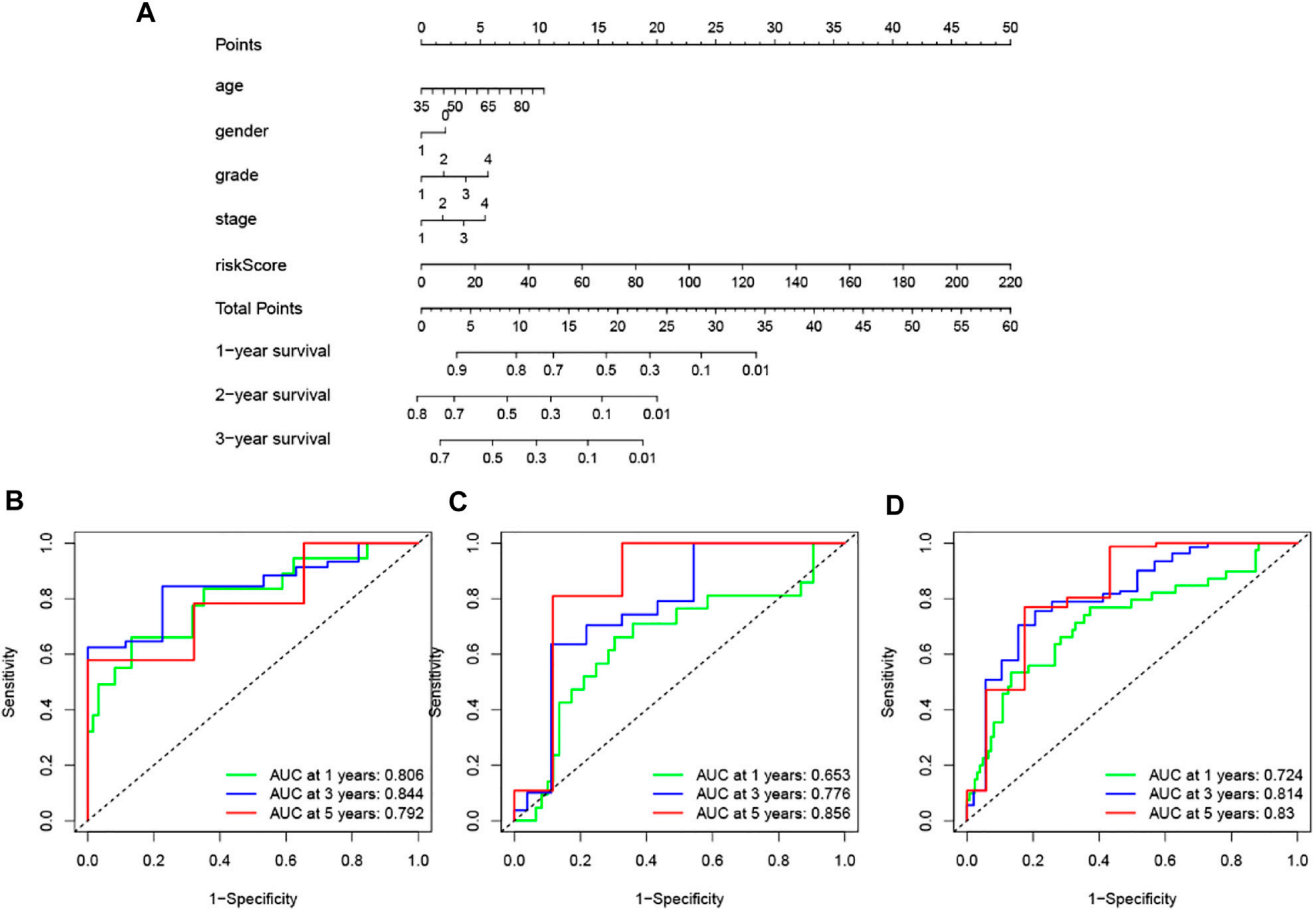
Construction and assessment of a nomogram for survival prediction of patients with pancreatic cancer based on risk score, age, gender, pathological grade and stage. **(A)** The nomogram was established in a training set for predicting 1-, 2-, and 3-years survival of pancreatic cancer patients. **(B–D)** ROC curve analysis for 1-, 2-, and 3-years survival prediction of the nomogram in the training set **(B)**, testing set **(C)**, and TCGA set **(D)**, respectively.

To improve the clinical application of the GILncSig, we established a prognostic nomogram model combined with the risk score, age, gender, pathological grade and stage to predict the patients’ survival at 1-, 2-, and 3- years in the training set by using “rms” and “survival” packages in software R (Figure 10A). In Figure 10B, the C-index of the nomogram of the training set was 0.650, and the AUC values predicted for 1-, 2- and 3-years survival is 0.806, 0.844, and 0.792, respectively. The C-index was 0.615 in the testing set and the AUCs of ROC for 1-, 2-, and 3-years survival predictions were 0.653, 0.776, and 0.856, respectively (Figure 10C). Likewise, the C-index was 0.618 in the whole TCGA set and the 1-, 2-, and 3-years AUCs were 0.724, 0.814, and 0.83, respectively (Figure 10D). The calibration plots in (Supplementary Figure 1) exhibited excellent accordance between the nomogram prediction and the actual values in terms of the 1-, 2- and 3-years survival rates in the three datasets. The above results indicated that the prediction performance of the established nomogram is improved.

### Correlation of risk score with tumor immune environment characterization

**FIGURE 11:**
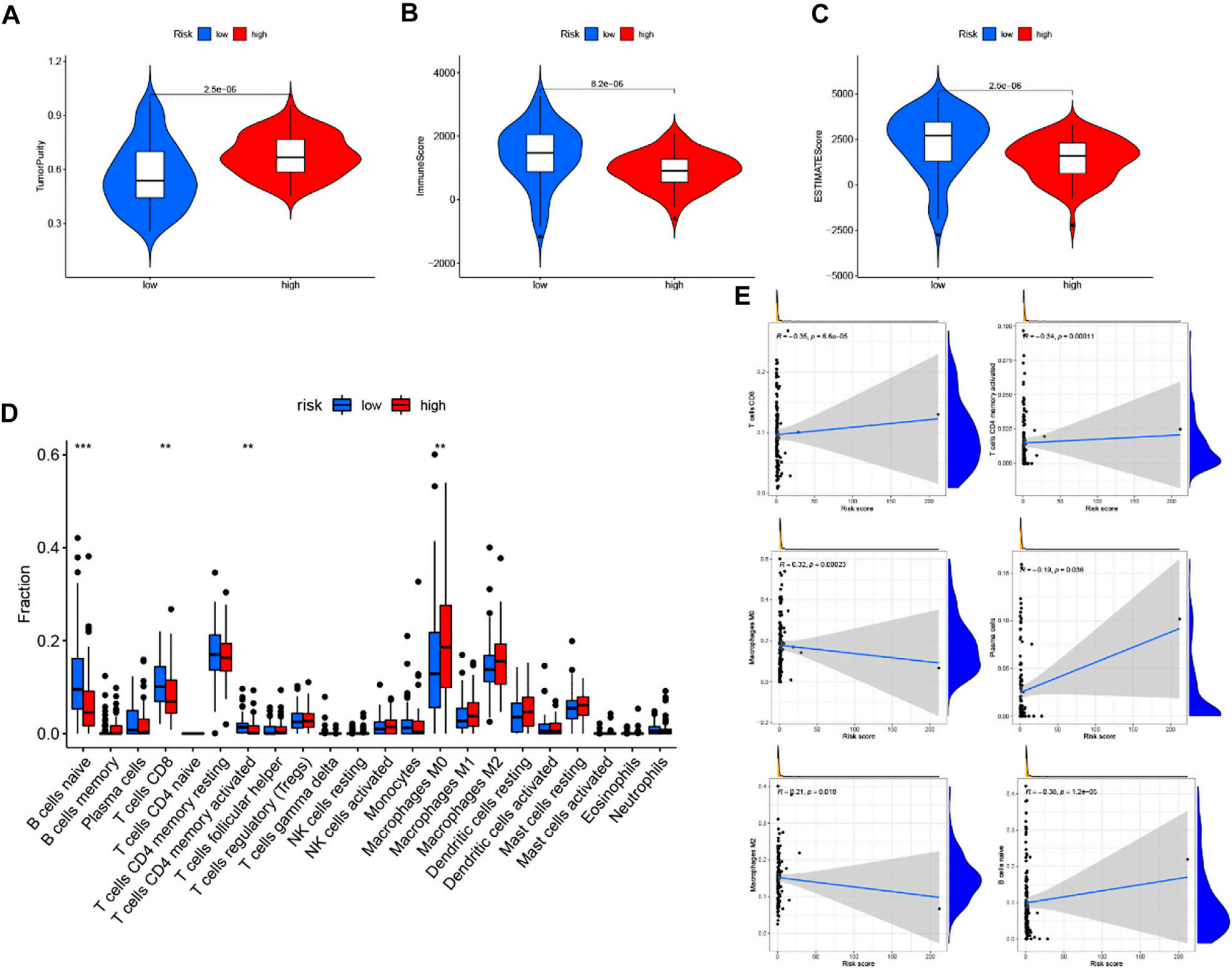
Correlation of prognostic risk score with TIME characterization **(A–C)** The correlation of estimate score, immune score, and tumor purity between these two subtypes. **(D)** Difference of infiltrating immune cell subpopulations and levels between low-/high-risk groups. **(E)** Correlation between tumor immune infiltration and GILncSig.

**FIGURE 12:**
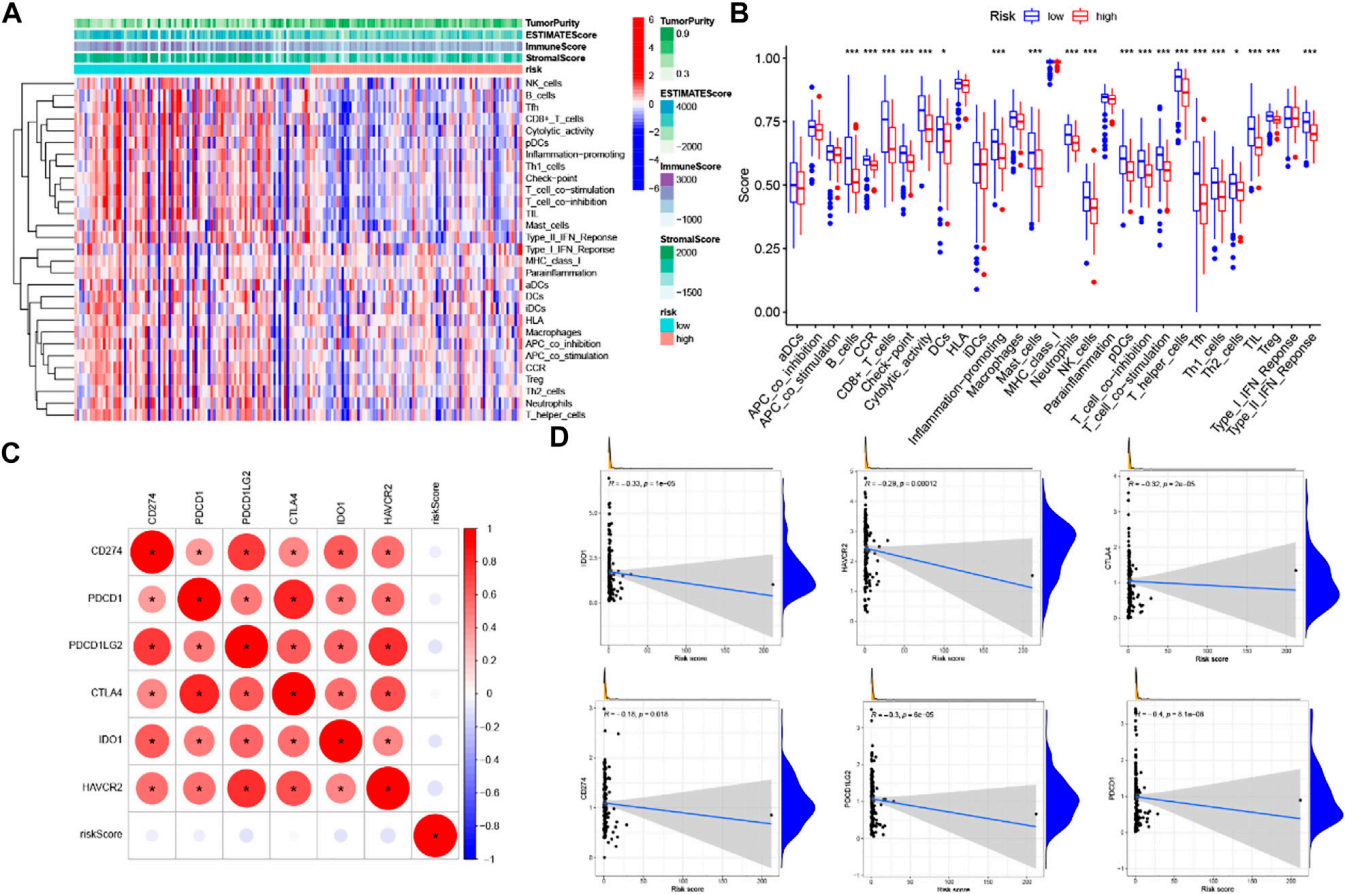
Correlation of prognostic risk score with TIME characterization **(A)** immune-related signature with corresponding immune-related scores in groups low/high risk. **(B)** A distinction of enrichment of immune-related signatures between low-/high-risk groups. **(C)** Association analyses between immune checkpoint inhibitors and GILncSig. **(D)** Association between risk model and CD274, CTLA4, HAVCR2, PDCD1, PDCD1LG2.

Through the ESTIMATE evaluation method, TumorPurity, ImmuneScore and StromalScore were calculated. These results indicated that patients in the low-risk group have lower TumorPurity and higher ImmuneScore and StromalScore (Figures 11A–C). To further uncover the correlation between GILncSig and immune cell infiltration, the analysis showed that patients in the low-risk group had more T cells CD8, B cells, and T cells CD4 memory activated, while the Macrophages M0 was at a low level (Figure 11D). To further explore the influence of GILncSig on the TIME of PC we analyzed the correlation of risk signature with immune cell infiltration type and level. The results indicated that the risk signature significantly correlated with infiltrating B cells (*r* = −0.38; *p* = 1.2*e* − 05), infiltrating CD4+T cells (*r* = −0.34; *p* = 0.00011), infiltrating plasma cells (*r* = −0.19; *p* = 0.036), CD8 T cells (*r* = -0.35; *p* = 6.6*e* −05), macrophages M0 (*r* = 0.32; *p* = 0.00023), and macrophages M2 (*r* = 0.21; *p* = 0.018; Figure 11E). Then, the ssGSEA algorithm was used to examine whether there was a distinction of immune signatures between groups of low/high risk. The results found that the infiltrating levels of B cells, CD8+T cells, DCs, Neutrophils, pDCs, Tfh, Th1 cells, and Th2 cells were remarkably elevated and some immune signatures (i.e., CCR, checkpoint, inflammation-promoting, IFN response type II) were significantly activated in the low-risk group Figure 12A,B).

### Correlation of risk score with immune checkpoint blockade key molecules

**FIGURE 13:**
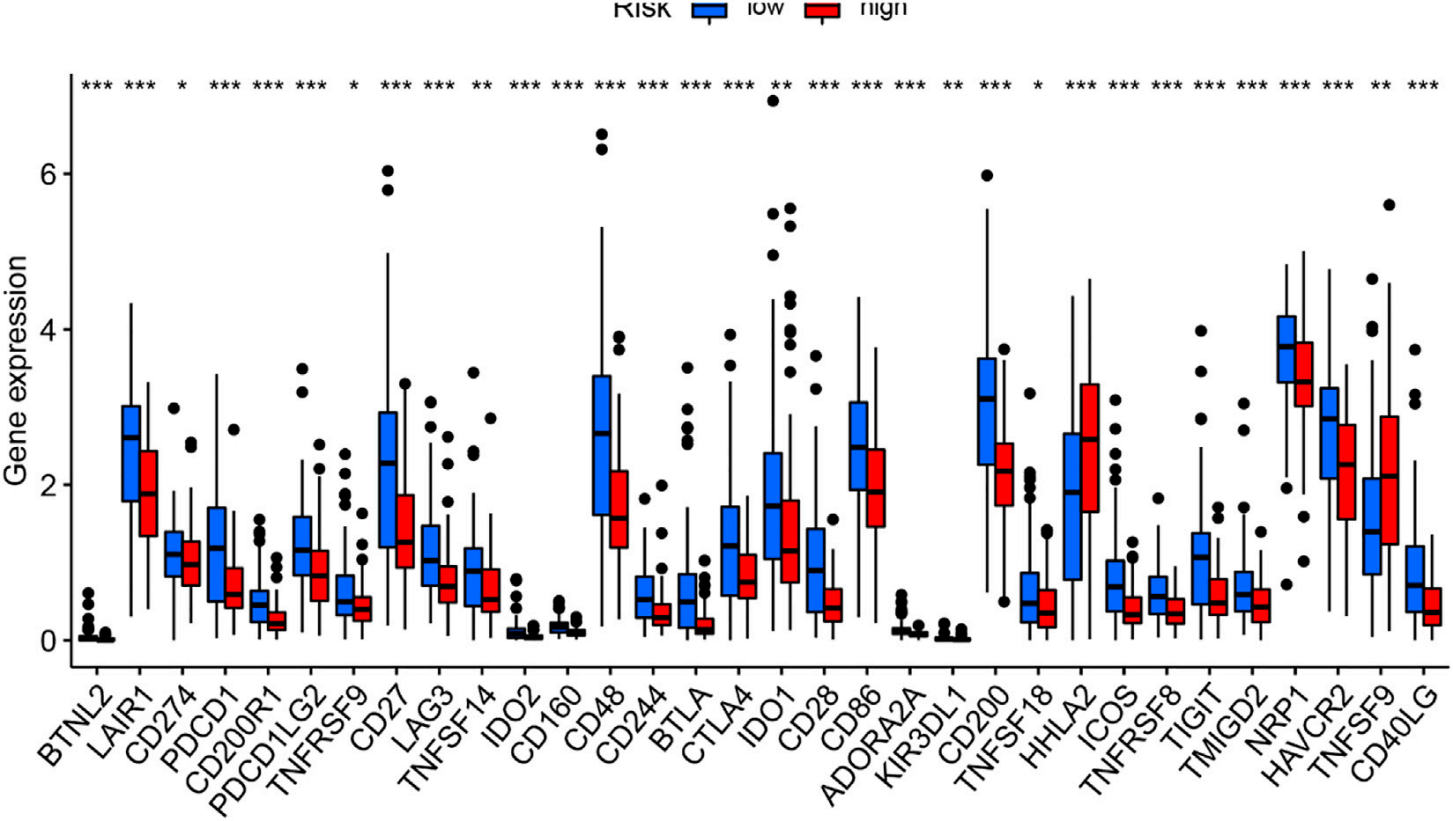
Comparison of 32 immune checkpoint blockade–related gene expression levels in low-/high-risk groups.

Six key immune checkpoint inhibitor genes (PDCD1, CD274, PDCD1LG2, CTLA‐4, HAVCR2, and IDO1) were singled out for further research. We performed the correlation analysis of ICB key gene expression with risk signature to investigate the potential role of a signature in the ICB therapy of PC (Figure 12C). Correlation analysis results indicated that GILncSig had close relationship with CD274 (*r* = −0.18; *p* = 0.018), CTLA4 (*r* = -0.32; p = 2e −05), HAVCR2 (*r* = -0.29; *p* = 0.00012), PDCD1 (i = −0.4; *p* = 8.1e − 08), and PDCD1LG2 (*r* = −0.3; p = 6e−05; Figures 12D), indicating GILncSig might exert a nonnegligible player in ICB treatment outcome prediction in PC. Further correlation analysis presented that 32 of 47 (i.e., CD27, IDO2, etc.) immune check blockade–related gene expression levels were significantly different between the two risk groups (Figures 13).

## Discussion

PC is the most common cause of cancer death worldwide (Siegel et al., 2020). It is characterized by high morbidity, high mortality, difficult early diagnosis, and poor prognosis (Sharma et al., 2011; Peng et al., 2016). Surgical resection is effective for patients with early PC, while palliative treatment is adopted for patients with locally advanced, metastatic, and unresectable PC (Li et al., 2004). In recent years, molecular research on PC has made great progress, and the survival rate of PC patients has improved to some extent. However, the prognosis has not been improved (Feldmann and Maitra, 2008). As metastasis and recurrence are the main causes of poor prognosis, it is urgent to identify effective tumor biomarkers to evaluate the prognosis of patients with PC accurately.

Genomic instability is an important feature of human cancer, which is associated with poor prognosis, and metastasis (Bakhoum and Cantley, 2018; Duijf et al., 2019). It has been reported that genomic instability affects the prognosis of PC, and the pattern of genomic instability is quite heterogeneous in metastatic PC (Campbell et al., 2010; Sahin et al., 2016). It is known that the degree of genomic instability has diagnostic and prognostic implications, yet measuring genomic instability is a big challenge. Mettu et al. (2010) constructed a 12-gene signature to assess genomic instability and predict clinical outcomes in cancers. Zhang et al.(2014) developed a biological rationale-driven genomic instability score to predict the prognosis of ovarian cancer.

LncRNAs have complex biological functions and have been proved to be closely related to the occurrence and development of cancers (Gibb et al., 2011; Fatica and Bozzoni, 2014). Recently, increasingly more researchers pay attention to the clinical significance of LncRNAs in the prognosis of cancers. For instance, the high expression of lncRNA HOX transcript antisense RNA (HOTAIR) in lung tumor tissues is correlated with metastasis and poor prognosis in patients with lung cancer (Loewen et al., 2014). The lncRNA AOC4P induces a poor prognosis in gastric cancer patients through epithelial-mesenchymal transition (Zhang et al., 2019). It has been found that lncRNAs play an important role in maintaining genomic stability through continuous exploration of the function of lncRNAs (Lee et al., 2016; Liu, 2016; Hu et al., 2018). Although some efforts have been made, few kinds of research have been done on GILnc in cancers. Therefore, there is an urgent need to investigate the prognostic value of genomic-instability associated lncRNAs in PC patients.

In our study, we identified 40 genomic instability-associated lncRNAs by analyzing the lncRNA expression profile and somatic mutation profile of 171 patients with PC. Then, the function of these lncRNAs was predicted by the lncRNA-mRNA co-expression network. The GO and KEGG enrichment results suggested that the genes co-expressed with these 206 lncRNAs were enriched at chromosomes and nucleoplasm in the cellular component, DNA binding in the molecular function, and the transcription and compound synthesis and metabolism in the biological process can promote genomic instability, which leads to cancer eventually (Barnum and O'Connell, 2014; Friedberg, 2001). We further divided all patients into the training set and testing set. Cox proportional risk regression analysis was performed on the candidate genomic instability-associated lncRNAs in the training set, and a genomic instability-associated lncRNAs signature (GILncSig) consisting of 5 lncRNAs with independent prognostic value (AL121772.1, BX640514.2, LINC01133, AC087752.3, and LYPLAL1-AS1) was established to predict the prognosis of PC. The GILncSig can classify PC patients in the training set into the high-risk group and low-risk group with significantly different overall survival, which was verified in the testing set and the whole TCGA set. In addition, we also found that patients with PC in the high-risk group had significantly higher somatic mutation counts and UBQLN4 expression levels, both of which are characteristics of genomic instability. A comparison of our GILncSig and two recently reported lncRNA-related signatures with predictive values for PC in the same TCGA patient set suggested that the GILncSig has better prognostic ability in predicting survival than those two lncRNA-related signatures. Our study also found that the GILncSig was independent of other clinicopathological factors, including age, gender, pathological grade, and stage. Furthermore, based on the GILncSig, the mutation status of KRAS, TP53, and SMAD4 in the high-risk group were significantly higher than those in the low-risk group. The survival time of KRAS, TP53, and SMAD4 wild-type patients in the low-risk group was significantly longer than that of patients with mutant-type.

The above results indicated that the GILncSig may have greater prognostic significance than KRAS, TP53 and SMAD4 mutation states. Finally, a nomogram was constructed by combining GInLncSig and the four independent prognostic factors of age, gender, pathological grade, and stage in the training set, which further improved the predictive performance, and was verified on the testing set and the entire TCGA set.

What’s more, numerous researches focusing on TIME have revealed the potential key role of lncRNAs in infiltrating immune cells. In this study, we find that GILncSig was significantly correlated with immune cell infiltration, ESTIMATE results showed that GILncSig was positive with tumor purity but negatively correlated with estimate score and immune score, suggesting GILncSig could serve as a novel immune indicator in PC. Besides, ssGSEA results indicated that in the low-risk group the infiltrating immune cells were significantly increased and immune signatures were remarkably activated. The immune-activated condition in the low-risk group was associated with high ICB-relevant gene expression, suggesting samples with high-risk scores might respond to immunotherapy. What’s more, the correlation analysis between ICB-related genes and GILncSig indicated that our signature may possess the ability to predict the clinical outcome of ICB therapy in PC.

Although the GILncSig identified here is reliable and promising as a prognostic signature in the tumor immune microenvironment of PC, there are still several limitations. In addition to validation in the TCGA dataset, the GILncSig requires more independent datasets to verify. Meanwhile, it is necessary to further explore the regulatory mechanism of GILncSig in biological function to maintain genomic instability.

## Conclusion

In summary, we have performed RNA-seq prognostic analysis in PC patients by bioinformatics methods to develop a genomic instability-derived lncRNA signature to predict the prognosis of PC patients and successfully validated it on the independent cohort. Moreover, we integrated GInLncSig with age, gender, pathological grade and stage to construct a nomogram to improve its prediction performance. Further results unraveled that GILncSig was significantly correlated with immune cell infiltration and has important significance for genomic instability and ICB treatment of PC.

## Data Availability

The original contributions presented in the study are included in the article/Supplementary Material, further inquiries can be directed to the corresponding authors.

## References

[B1] Bakhoum S. F. Cantley L. C. (2018). The multifaceted role of chromosomal instability in cancer and its microenvironment. Cell 174 (6), 1347–1360. 10.1016/j.cell.2018.08.027 30193109 PMC6136429

[B2] Barnum K. J. O'Connell M. J. (2014). Cell cycle regulation by checkpoints. Methods Mol. Biol. 1170, 29–40. 10.1007/978-1-4939-0888-2_2 24906307 PMC4990352

[B3] Braconi C. Kogure T. Valeri N. Huang N. Nuovo G. Costinean S. (2011). microRNA-29 can regulate expression of the long non-coding RNA gene MEG3 in hepatocellular cancer. Oncogene 30 (47), 4750–4756. 10.1038/onc.2011.193 21625215 PMC4292930

[B4] Campbell P. J. Yachida S. Mudie L. J. Stephens P. J. Pleasance E. D. Stebbings L. A.. (2010). The patterns and dynamics of genomic instability in metastatic pancreatic cancer. Nature 467 (7319), 1109–1113. 10.1038/nature09460 20981101 PMC3137369

[B5] Cheetham S. W. Gruhl F. Mattick J. S. Dinger M. E. (2013). Long noncoding RNAs and the genetics of cancer. Br. J. Cancer 108 (12), 2419–2425. 10.1038/bjc.2013.233 23660942 PMC3694235

[B6] Diao P. Song Y. Ge H. Wu Y. Li J. Zhang W. (2019). Identification of 4-lncRNA prognostic signature in head and neck squamous cell carcinoma. J. Cell. Biochem. 120 (6), 10010–10020. 10.1002/jcb.28284 30548328

[B7] Duijf P. H. G. Nanayakkara D. Nones K. Srihari S. Kalimutho M. Khanna K. K. (2019). Mechanisms of genomic instability in breast cancer. Trends Mol. Med. 25 (7), 595–611. 10.1016/j.molmed.2019.04.004 31078431

[B8] Fatica A. Bozzoni I. (2014). Long non-coding RNAs: New players in cell differentiation and development. Nat. Rev. Genet. 15 (1), 7–21. 10.1038/nrg3606 24296535

[B9] Fei Y. Gao K. Tu J. Wang W. Zong G. Q. Li W. Q. (2018). Predicting and evaluation the severity in acute pancreatitis using a new modeling built on body mass index and intra-abdominal pressure. Am. J. Surg. 216 (2), 304–309. 10.1016/j.amjsurg.2017.04.017 28888465

[B10] Feldmann G. Maitra A. (2008). Molecular genetics of pancreatic ductal adenocarcinomas and recent implications for translational efforts. J. Mol. Diagn. 10 (2), 111–122. 10.2353/jmoldx.2008.070115 18258927 PMC2259464

[B11] Friedberg E. C. (2001). How nucleotide excision repair protects against cancer. Nat. Rev. Cancer 1 (1), 22–33. 10.1038/35094000 11900249

[B12] Garcea G. Neal C. P. Pattenden C. J. Steward W. P. Berry D. P. (2005). Molecular prognostic markers in pancreatic cancer: A systematic review. Eur. J. Cancer 41 (15), 2213–2236. 10.1016/j.ejca.2005.04.044 16146690

[B13] Garrido-Laguna I. Hidalgo M. (2015). Pancreatic cancer: From state-of-the-art treatments to promising novel therapies. Nat. Rev. Clin. Oncol. 12 (6), 319–334. 10.1038/nrclinonc.2015.53 25824606

[B14] Gibb E. A. Brown C. J. Lam W. L. (2011). The functional role of long non-coding RNA in human carcinomas. Mol. Cancer 10, 38. 10.1186/1476-4598-10-38 21489289 PMC3098824

[B15] Gupta R. Sinha S. Paul R. N. (2018). The impact of microsatellite stability status in colorectal cancer. Curr. Probl. Cancer 42 (6), 548–559. 10.1016/j.currproblcancer.2018.06.010 30119911

[B16] Hauptman N. Glavac D. (2013). Long non-coding RNA in cancer. Int. J. Mol. Sci. 14 (3), 4655–4669. 10.3390/ijms14034655 23443164 PMC3634483

[B17] Hu W. L. Jin L. Xu A. Wang Y. F. Thorne R. F. Zhang X. D. (2018). GUARDIN is a p53-responsive long non-coding RNA that is essential for genomic stability. Nat. Cell Biol. 20 (4), 492–502. 10.1038/s41556-018-0066-7 29593331

[B18] Lee S. Kopp F. Chang T. C. Sataluri A. Chen B. Sivakumar S. (2016). Noncoding RNA NORAD regulates genomic stability by sequestering PUMILIO proteins. Cell 164 (1-2), 69–80. 10.1016/j.cell.2015.12.017 26724866 PMC4715682

[B19] Li D. Xie K. Wolff R. Abbruzzese J. L. (2004). Pancreatic cancer. Lancet (London, Engl. 363 (9414), 1049–1057. 10.1016/S0140-6736(04)15841-8 15051286

[B20] Lin T. Fu Y. Zhang X. Gu J. Ma X. Miao R.. (2018). A seven-long noncoding RNA signature predicts overall survival for patients with early stage non-small cell lung cancer. Aging 10 (9), 2356–2366. 10.18632/aging.101550 30205363 PMC6188476

[B21] Liu H. (2016). Linking lncRNA to genomic stability. Sci. China. Life Sci. 59 (3), 328–329. 10.1007/s11427-016-5009-6 26780343

[B22] Loewen G. Jayawickramarajah J. Zhuo Y. Shan B. (2014). Functions of lncRNA HOTAIR in lung cancer. J. Hematol. Oncol. 7, 90. 10.1186/s13045-014-0090-4 25491133 PMC4266198

[B23] Ma L. Bajic V. B. Zhang Z. (2014). On the classification of long non-coding RNAs. RNA Biol. 10 (6), 925–933. 10.4161/rna.24604 PMC4111732 23696037

[B24] McGuigan A. Kelly P. Turkington R. C. Jones C. Coleman H. G. McCain R. S. (2018). Pancreatic cancer: A review of clinical diagnosis, epidemiology, treatment and outcomes. World J. Gastroenterol. 24 (43), 4846–4861. 10.3748/wjg.v24.i43.4846 30487695 PMC6250924

[B25] Mercer T. R. Dinger M. E. Mattick J. S. (2009). Long non-coding RNAs: Insights into functions. Nat. Rev. Genet. 10 (3), 155–159. 10.1038/nrg2521 19188922

[B26] Mettu R. K. Wan Y. W. Habermann J. K. Ried T. Guo N. L. (2010). A 12-gene genomic instability signature predicts clinical outcomes in multiple cancer types. Int. J. Biol. Markers 25 (4), 219–228. 10.5301/jbm.2010.6079 21161944 PMC3155635

[B27] Peng J-F. Zhuang Y. Y. Huang F. T. Zhang S. N. (2016). Noncoding RNAs and pancreatic cancer. World J. Gastroenterol. 22 (2), 801–814. 10.3748/wjg.v22.i2.801 26811626 PMC4716078

[B28] Rinn J. L. Chang H. Y. (2012). Genome regulation by long noncoding RNAs. Annu. Rev. Biochem. 81 (1), 145–166. 10.1146/annurev-biochem-051410-092902 22663078 PMC3858397

[B29] Sahin I. H. Lowery M. A. Stadler Z. K. Salo-Mullen E. Iacobuzio-Donahue C. A. Kelsen D. P. (2016). Genomic instability in pancreatic adenocarcinoma: A new step towards precision medicine and novel therapeutic approaches. Expert Rev. Gastroenterol. Hepatol. 10 (8), 893–905. 10.1586/17474124.2016.1153424 26881472 PMC4988832

[B30] Sharma C. Eltawil K. M. Renfrew P. D. Walsh M. J. Molinari M. (2011). Advances in diagnosis, treatment and palliation of pancreatic carcinoma: 1990-2010. World J. Gastroenterol. 17 (7), 867–897. 10.3748/wjg.v17.i7.867 21412497 PMC3051138

[B31] Shi X. Zhao Y. He R. Zhou M. Pan S. Yu S.. (2018). Three-lncRNA signature is a potential prognostic biomarker for pancreatic adenocarcinoma. Oncotarget 9 (36), 24248–24259. 10.18632/oncotarget.24443 29849937 PMC5966255

[B32] Siegel R. L. Miller K. D. Jemal A. (2020). Cancer statistics, 2020. Ca. Cancer J. Clin. 70 (1), 7–30. 10.3322/caac.21590 31912902

[B33] Song J. Xu Q. Zhang H. Yin X. Zhu C. Zhao K. (2018). Five key lncRNAs considered as prognostic targets for predicting pancreatic ductal adenocarcinoma. J. Cell. Biochem. 119 (6), 4559–4569. 10.1002/jcb.26598 29239017 PMC5947154

[B34] Spizzo R. Almeida M. I. Colombatti A. Calin G. A. (2012). Long non-coding RNAs and cancer: A new frontier of translational research? Oncogene 31 (43), 4577–4587. 10.1038/onc.2011.621 22266873 PMC3433647

[B35] Tang J. Ren J. Cui Q. Zhang D. Kong D. Liao X. (2019). A prognostic 10-lncRNA expression signature for predicting the risk of tumour recurrence in breast cancer patients. J. Cell. Mol. Med. 23 (10), 6775–6784. 10.1111/jcmm.14556 31429520 PMC6787455

[B36] Troiano G. Caponio V. C. A. Boldrup L. Gu X. Muzio L. L. Sgaramella N. (2017). Expression of the long non-coding RNA HOTAIR as a prognostic factor in squamous cell carcinoma of the head and neck: A systematic review and meta-analysis. Oncotarget 8 (42), 73029–73036. 10.18632/oncotarget.20373 29069846 PMC5641189

[B37] Wang J. Z. Xiang J. J. Wu L. G. Bai Y. S. Chen Z. W. Yin X. Q.. (2017). A genetic variant in long non-coding RNA MALAT1 associated with survival outcome among patients with advanced lung adenocarcinoma: A survival cohort analysis. BMC Cancer 17 (1), 167. 10.1186/s12885-017-3151-6 28253859 PMC5335789

[B38] Xiaole Z. Rong Y. Yunpeng P. Chaoqun H. Chenyuan S. Lingdi Y. (2021). Acta chimica sinica. Research Square.

[B39] Yu G. Wang L-G. Han Y. He Q-Y. (2012). clusterProfiler: an R Package for comparing biological themes among gene clusters. OMICS A J. Integr. Biol. 16 (5), 284–287. 10.1089/omi.2011.0118 PMC3339379 22455463

[B40] Zhang K. Lu C. Huang X. Cui J. Li J. Gao Y. (2019). Long noncoding RNA AOC4P regulates tumor cell proliferation and invasion by epithelial-mesenchymal transition in gastric cancer. Ther. Adv. Gastroenterol. 12, 1756284819827697. 10.1177/1756284819827697 PMC6383096 30815034

[B41] Zhang S. Yuan Y. Hao D. (2014). A genomic instability score in discriminating nonequivalent outcomes of BRCA1/2 mutations and in predicting outcomes of ovarian cancer treated with platinum-based chemotherapy. PloS one 9 (12), e113169. 10.1371/journal.pone.0113169 25437005 PMC4249855

[B42] Zhang Y. Shields T. Crenshaw T. Hao Y. Moulton T. Tycko B. (1993). Imprinting of human H19: Allele-specific CpG methylation, loss of the active allele in wilms tumor, and potential for somatic allele switching. Am. J. Hum. Genet. 53 (1), 113–124. 8391213 PMC1682243

[B43] Zhou M. Sun Y. Sun Y. Xu W. Zhang Z. Zhao H. (2016). Comprehensive analysis of lncRNA expression profiles reveals a novel lncRNA signature to discriminate nonequivalent outcomes in patients with ovarian cancer. Oncotarget 7 (22), 32433–32448. 10.18632/oncotarget.8653 27074572 PMC5078024

