# Supplementary material for: Identification of genomic instability related lncRNA signature with prognostic value and its role in cancer immunotherapy in pancreatic cancer
Source: Front Genet. 2022 Sep 2;13:990661. doi: 10.3389/fgene.2022.990661 (PMC9481284; doi:10.3389/fgene.2022.990661)
Supplement: Supplementary file 1 [file Image1.pdf]

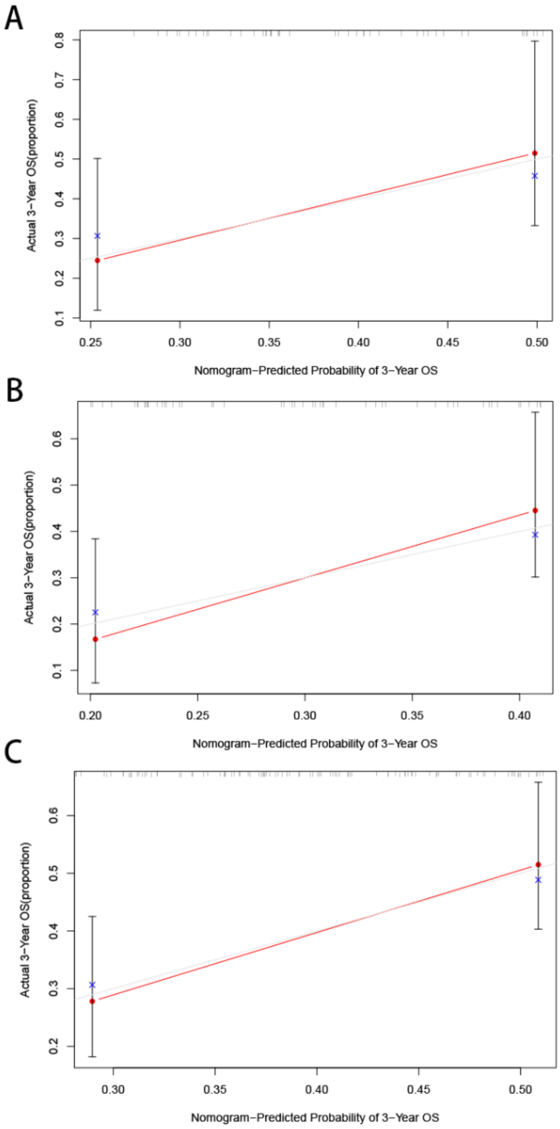

Supplementary Figure 1: The calibration plots of the survival prediction for actual survival rate and predictions in the training set (A), testing set (B), and TCGA set (C), respectively.
